# Pharmacy Students’ Mental Health and Resilience in COVID-19: An Assessment after One Year of Online Education

**DOI:** 10.3390/ejihpe12080077

**Published:** 2022-08-15

**Authors:** Dalal Hammoudi Halat, Samar Younes, Jihan Safwan, Zeina Akiki, Marwan Akel, Mohamad Rahal

**Affiliations:** 1 Pharmaceutical Sciences Department, School of Pharmacy, Lebanese International University, Bekaa P.O. Box 146404, Lebanon; 2 Biomedical Sciences Department, School of Pharmacy, Lebanese International University, Bekaa P.O. Box 146404, Lebanon; 3 Faculty of Public Health, Lebanese University, Beirut 6573, Lebanon; 4 INSPECT-LB: Institut National de Santé Publique, Épidémiologie Clinique et Toxicologie-Liban, Beirut 6573, Lebanon; 5 Pharmacy Practice Department, School of Pharmacy, Lebanese International University, Beirut P.O. Box 146404, Lebanon

**Keywords:** COVID-19, mental health, pharmacy students, pharmacy education, DASS-21, BRS

## Abstract

COVID-19 has impacted mental health and affected education due to the shift to remote learning. The purpose of the current study was to assess the mental health of pharmacy students one year following the onset of the pandemic. A descriptive cross-sectional questionnaire was distributed to pharmacy students. The severity of depression, anxiety, and stress was assessed by the Depression Anxiety Stress Scale (DASS-21), and resilience was assessed by the Brief Resilience Scale (BRS). COVID-19-related economic, educational, and health stressors, and students’ vaccine attitudes were surveyed. Descriptive, bivariate, and multivariable analysis were used, and a *p*-value of <0.05 was considered significant. A total of 561 students participated; 37% had mild-to-moderate depression, 37% had severe-to-extremely-severe anxiety, and 52% demonstrated normal stress levels. Severe depression, anxiety, and stress were associated with smoking and feeling isolated due to COVID-19. Around 40% of students had low resilience, associated with smoking, being in the third or fourth year of pharmacy study, and the consumption of caffeinated beverages. The mean score of satisfaction with online learning was 60.3 ± 21.3%. Only 5% of participants were vaccinated, of which 87% trusted the benefits of vaccines and their role in controlling the pandemic. One year after the onset of COVID-19, depression, anxiety, stress, and low resilience were observed among pharmacy students; the investigation of the long-term mental effects of the pandemic on university students is warranted.

## 1. Introduction

Since December 2019, the outbreak caused by the severe acute respiratory distress syndrome coronavirus 2 (SARS-CoV-2) has spread to different parts of the world, causing an emerging respiratory illness with serious complications [1]. Soon thereafter, the associated disease, termed coronavirus disease 2019 (COVID-19), was labeled as a pandemic by the World Health Organization (WHO) [2]. The pandemic continues to affect the lives of hundreds of millions of patients and to have profound effects on society, the environment, the economy, politics, and global biosafety as a whole [3,4]. Moreover, the WHO has expressed concerns over the pandemic’s mental health and psychosocial consequences, speculating that the required measures of self-isolation and distancing have affected the usual activities, routines, and livelihoods of people, generating numerous mental issues [5]. According to the literature, the pandemic is raising distress, panic, depressive symptoms, obsessive–compulsive disorder, post-traumatic stress disorder, and anxiety in individuals exposed to the peril of the virus across the globe [6,7]. Furthermore, the psychosocial effects of quarantine, required from patients and their contacts, were reported as long-lasting, with fear, frustration, boredom, inadequate information, financial loss, and stigma negatively deploying mental status and representing another dark side of COVID-19 [8].

In addition to impacts on mental health, the pandemic has brought unprecedented stress to the educational system, including universities and their students. After a shift to remote instruction, higher education has faced the difficult decision of whether to reopen in the 2020–2021 academic year and risk events of viral super spreading, or to keep classes online in the face of potential losses of revenue and a diminished ability to support vulnerable students [9]. As such, the pandemic has exposed students to exceptional fluctuations and uncertainties, starting with the initial transition to online instruction in 2020, and further exacerbated by a long summer of social isolation, lost employment, and ambiguity about the structure of courses and university arrangements in the 2020–2021 academic year [10,11], the second year for education to witness the effects of the pandemic. The intensive measures taken globally by universities and the sudden norm prompted by the pandemic are expected to affect the mental health and well-being of students [12]. Reports after the onset of the pandemic described acute stress, anxiety, depressive symptoms, and suicidal thoughts among university students, with multiple correlating epidemic and psychosocial factors, such as infection among family members, massive media exposure, low social support, senior study years, and prior mental health problems [13,14,15]. Students reported a number of academic and everyday difficulties and high levels of mental health distress associated with an inability to focus on academic work and elevated concern regarding COVID-19 [16]. Furthermore, a high prevalence of mental health issues were documented among students who experienced quarantine, underlining the need to reinforce prevention, surveillance, and access to care in this population [17].

In Lebanon, the first case of COVID-19 was officially confirmed on 21 February 2020. On 15 March 2020, and in an effort to contain the transmission of the disease, the Lebanese government imposed a general lockdown on different sectors of the country including schools, clubs, restaurants, businesses, places of worship, as well as the closure of the airport and the suspension of all travel [18]. Messages from the Ministry of Public Health were circulated, asking individuals to practice social distancing, self-isolation, and proper hygiene. Updates on the COVID-19 status in Lebanon were made available to the general public through the Ministry’s official website, mobile application, and social media pages. Additionally, press conferences were held to issue important notices and communicate the pandemic changes on a national level. After reevaluating the situation, the government extended the initial lockdown on April 9th for two additional weeks, extended again repeatedly until the end of May 2020; a status of uncertainty and interrupted lockdowns persisted thereafter for almost one year within the pandemic [19]. As of July 2022, the cumulative number of COVID-19 cases in Lebanon exceeds 1 million, and the disease has claimed the lives of over 10,500 individuals, while vaccination rates are close to 26% [20].The aforementioned lockdown of course extended to universities as well, and our university rapidly shifted to an online mode of education, which has continued since the spring semester of the academic year 2019–2020 to the present day [21,22]. Likewise, at the School of Pharmacy, pedagogical activities were shifted to an online mode, and all aspects of didactic courses, experiential education, laboratory work, and assessments were shifted to a remote structure, with some hybrid activities [22]. Accompanying this curricular modification, a temporary suspension or postponing of most extracurricular activities and events was enacted, as well as the moving of all advising actions and services to electronic mode, collectively resulting in a major shift in the educational structure for students [23]. Prior to the pandemic, studies assessing the mental health of pharmacy students have revealed high academic stress levels and poor mental health [24,25], sometimes even higher than other students [26]. In the wake of the global pandemic, studies from this region [27,28] and elsewhere [29,30] have demonstrated mental health concerns and negative feelings among pharmacy students, highlighting the need for proper educational reforms and approaches to improve students’ quality of life during times of vulnerability. As such, a local study that assesses Lebanese pharmacy students’ mental health was deemed necessary.

The mental health of the Lebanese population during the pandemic was previously assessed, indicating significant psychological stress, depression, anxiety, and obsessive–compulsive traits [31,32,33]. On top of the pandemic, Lebanon is facing severe socio-economic collapse and political turmoil as of October 2019, with the combined effects of COVID-19 and economy-related variables on mental health in a developing and crises-stricken country being well described [34]. It may be hypothesized, therefore, that the mental health of pharmacy students in Lebanon, given the educational changes due to COVID-19 and the country’s compounded crises, may have been unfavorably affected, with probable altered levels of depression, anxiety, stress, and resilience. However, to our knowledge, no previous survey has assessed the mental health of Lebanese pharmacy students and the factors affecting it after the elapse of time wherein the pandemic lingered and online education matured into the new normal. The purpose of the current study was to explore the impact of COVID-19 on the mental health of our pharmacy students one year following the declaration of the pandemic and after almost four semesters of remotely delivered pharmacy education.

## 2. Materials and Methods

### 2.1. Participants

This study was conducted among pharmacy students studying at the Lebanese International University (LIU) during May 2021 of the spring semester of 2020–2021 almost one year after the declaration of the pandemic and four semesters of remote education.

According to the LIU’s records, the number of pharmacy students studying at the university during the spring semester of 2020–2021 was 1515. Accordingly, and with reference to Epi-info software, a minimum sample size of 307 pharmacy students was needed based on a 95% confidence level with a margin of error of ±5. A total of 561 students participated in the study and their demographic data are shown in Table 1.

### 2.2. Instruments

The structured questionnaire consisted of questions that covered several areas divided into five sections. The first section addressed the students’ sociodemographic data: age, gender, nationality, area of residence, marital status, household size, year in pharmacy school, and monthly family income. Additionally, students were asked about their lifestyle including their smoking status, body weight status, and their consumption of alcohol, caffeinated beverages, medications and energy drinks during the COVID-19 pandemic. In the second and third sections, two validated scales that served the purpose of the study in measuring the mental health status and resilience of pharmacy students during the COVID-19 pandemic were used. Then, sections related to COVID-19 stressors and vaccine attitudes were included as described below.

#### 2.2.1. Depression, Anxiety, and Stress Scale 21 Items (DASS-21)

The DASS-21 is a 21-item system that includes a set of three self-reported scales which provide independent measures of depression, stress, and anxiety, with recommended severity thresholds. Each of the three DASS-21 scales contains seven items, divided into subscales with similar content. The depression scale assesses dysphoria, hopelessness, the devaluation of life, self-deprecation, the lack of interest/involvement, anhedonia, and inertia. The anxiety scale assesses autonomic arousal, skeletal muscle effects, situational anxiety, and the subjective experience of anxious affect. The stress scale is sensitive to levels of chronic nonspecific arousal. It assesses difficulty relaxing, nervous arousal, and being easily upset/agitated, irritable/over-reactive, and impatient. Each of the 21 items comprises a statement and four short-response options to reflect severity, scored from 0 to 3 [35].

Students were asked to read each statement and choose a number, 0, 1, 2, or 3, which indicated how much the statement applied to them over the past week. The rating scale is as follows: 0 (Did not apply to me at all); 1 (Applied to me to some degree or some of the time); 2 (Applied to me to a considerable degree or a good part of time); 3 (Applied to me very much or most of the time). Scores for depression, anxiety and stress were calculated by summing the scores for the relevant items (Questions 3, 5, 10, 13, 16, 17, 21 for depression; Questions 1, 6, 8, 11, 12, 14, 18 for stress; Questions 2, 4, 7, 9, 15, 19, 20 for anxiety). The Cronbach’s alpha for the DASS-21 subscales were as follows: depression scale 0.886; anxiety scale 0.84; and stress scale 0.871, indicating a good internal consistency. The severity of the DASS-21 subscales were computed and expressed as normal, mild to moderate, and severe to extremely severe [36].

#### 2.2.2. Brief Resilience Scale (BRS)

Resilience is defined as the ability to maintain healthy levels of functioning despite difficult experiences, or to return to normal functioning after experiences of adversity. High levels of resilience are associated with being optimistic, acting positively, and representing self-assurance when experiencing difficult life situations; it is, therefore, linked to better physical and mental health and well-being. People with high resilience levels who experience serious threats and crises have more positive mental health outcomes and are described as being more flexible and more adaptive upon responding to crises [37].

The BRS is a simple self-assessment tool that is used to assess the perceived ability to bounce back or recover from stress. The scale was developed to assess a unitary construct of resilience, including both positively and negatively worded items. To complete the scale, the participants were asked to indicate the extent to which they agreed with each of the six items according to a 5-point rating scale. The BRS was scored by reverse-coding items 2, 4, and 6, and calculating the sum of all six items. This summing-up provided an overall resilience score between 6 and 30, where the possible score range on the BRS is from 1 (low resilience) to 5 (high resilience). A weighted score was then calculated by dividing the total score by the number of items; in this case, a higher score would be reflective of greater resilience. The Cronbach’s alpha for the BRS score was 0.5, indicating an acceptable internal consistency. BRS scores obtained were interpreted as follows: low resilience (1.00–2.99); normal resilience (3.00–4.30); and high resilience (4.31–5.00) [38].

#### 2.2.3. COVID-19 Stressors

Moreover, the fourth part of the survey assessed economic, educational, and health stressors due to COVID-19. First, students were asked whether they were employed and whether they or their parents had lost employment or experienced employment reduction during COVID-19. Second, educational stressors were measured by asking students to rate their satisfaction regarding distance learning at the School of Pharmacy during COVID-19 on a 5-point Likert scale ranging from 1 (strongly disagree) to 5 (strongly agree). Third, COVID-19 health stressors were assessed by asking students whether they or their close family members in the same household had been diagnosed with COVID-19 and reporting the severity of their symptoms and the need for hospitalization. Finally, students were asked whether they felt isolated from others due to COVID-19.

#### 2.2.4. Vaccine Attitudes

The final part of the survey assessed the students’ attitudes towards vaccines. First, participants were asked whether they had received any COVID-19 vaccine and if so, what type. Then, both vaccinated and non-vaccinated participants were asked whether they were worried about the side effects of the vaccines. Finally, they were questioned as to whether they trusted the benefits of COVID-19 vaccines and the role of vaccines in controlling the pandemic, and if they trusted the governmental plan regarding the distribution and administration of COVID-19 vaccines.

### 2.3. Procedure

A descriptive cross-sectional study was performed via an anonymous online questionnaire to address the study’s objectives. The investigators developed the online questionnaire in the English language by using Google Forms, since English is the language of instruction at the university. A pilot phase was conducted to test the online questionnaire before initiating the actual data collection. It was completed by 10 students and 5 faculty members who provided their feedback and recommendations regarding the survey, and their results were not included in the analysis. Accordingly, a final version of the survey was prepared by the research team based on the delivered recommendations and feedback. This final version was sent to the emails of all pharmacy students studying at LIU across the nine campuses in different geographic areas of Lebanon, and they were invited to participate in the study on a voluntary basis. The questionnaire link was sent via emails and Google Classroom, the platform used at LIU for online instruction, where all students benefit from a personal Gmail and Google Workspace. The study scope and purpose were explained at the beginning of the questionnaire. Participants were informed that their participation in the study was voluntary and they were assured that their responses would remain anonymous and confidential. The completion of the entire questionnaire was considered as informed consent to participate. The Research Committee of the School of Pharmacy at the Lebanese International University approved the study.

### 2.4. Data Analysis

Descriptive statistics were performed to represent the participants’ characteristics, COVID-19 related information, the DASS-21 score, the BRS score, COVID-19 stressors, and vaccine attitudes, and these were expressed as median (Q1;Q3) or percentages. The DASS-21 subscales were expressed as “mild to moderate” versus “normal”, and “severe to extremely severe” versus “normal”, and the BRS score as “normal to high” versus “low”. Moreover, the associations between the scores and the participants’ characteristics were assessed using the Chi-square test, Student’s *T*-test, ANOVA, or their non-parametric tests when applicable. Four binary logistic regression models were used to evaluate the association between the scores and the potential confounders. In the first, second, and third regressions, the depression DASS-21 subscale, the anxiety subscale, and the stress subscale were the dependent variables, respectively. In the fourth logistic regression, the BRS score was the dependent variable. The participants’ characteristics having a *p*-value of less than 0.05 in the bivariate analysis (such as age, gender, smoking, educational level, marital status, etc.) were included as covariates. The model was tested for adequacy in all the analyses.

**Figure 1:**
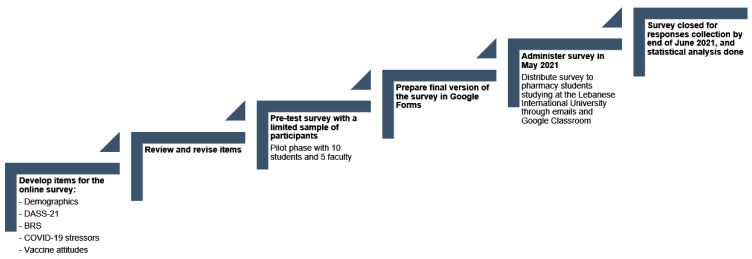
Flow chart describing the study’s steps and methodology. DASS21: Depression, Anxiety, and Stress Scale 21 Items; BRS: Brief Resilience Scale; COVID-19: Coronavirus Disease 2019.

An alpha of 0.05 was used to determine statistical significance. All analyses were performed using the IBM’s Statistical Package for the Social Sciences (SPSS) version 22.0 (IBM, Inc., Chicago, IL, USA). A flow chart showing the timeline and methodology of this study is presented in Figure 1.

## 3. Results

### 3.1. Sociodemographic Parameters

A total of 561 students completed the questionnaire; the demographic characteristics of participants and changes in their lifestyle during the pandemic are shown in Table 1. The majority of participants were Lebanese (95%) and female (76%), as is consistent with gender trends at the School of Pharmacy. Different geographic areas of Lebanon were represented among the participants, since the School of Pharmacy offers courses across eight campuses dispersed in different areas of the country. Only a minority (about 1%) of the participants were answering the questionnaire while enrolled at the school but residing abroad, an option possible with the spring semester of 2020–2021 being delivered almost completely online. Most of the participants were single (93%) and lived in a household with a family size ranging between three and five individuals (63%). Participants’ year of study was distributed among the five years of the Bachelor of Pharmacy program as well as the sixth year or Doctor of Pharmacy (PharmD).

### 3.2. Lifestyle Parameters and Variations during COVID-19

Almost 79% of the participants were nonsmokers, 45% consumed less than two daily caffeinated beverages, and 85% did not consume energy drinks. When asked about some habit changes during the pandemic, 86% answered that they did not consume alcoholic drinks and 39% reported weight loss during COVID-19. While the majority had a family income of 2,000,000–4,000,000 Lebanese pounds per month, the vast majority (73%) reported that their income was affected by the pandemic, while only 17% received financial support from abroad. Concerning medication intake during the pandemic, the answers of the participants ranged from 3% taking antidepressants and/or anxiolytics, to 30% taking multivitamins or minerals (Table 2).

### 3.3. Results of the DASS-21 and BRS Scales

Calculation of depression subscale from the DASS-21 items related to depression revealed that 37% of the participants had mild to moderate depression. Additionally, 37% had severe to extremely severe anxiety on the DASS-21 anxiety subscale, and 52% had normal stress levels on the DASS-21 stress subscale. Regarding the resilience of participants measured by the BRS, over 60% of participants had normal to high resilience. Details of both the DASS-21 and BRS are shown in Table 3.

### 3.4. Results of the Bivariate Analysis

In the bivariate analysis, multiple factors were significantly associated with depression, anxiety, and stress among pharmacy students. In the multivariable logistic regression models, those associations weakened but statistical difference still existed. Table 4 shows the association of possible influence factors one year following the declaration of the COVID-19 pandemic and after almost four semesters of remotely delivered pharmacy education. For example, in the depression subscale, severe or extremely severe depression was associated with a household of 1–2 persons, being a smoker or alcoholic, and the presence of reported changes in body weight. It was also significantly associated with taking medications including acetaminophen, antihistamines, Non-Steroidal Anti-Inflammatory Drugs (NSAIDs), multivitamins, minerals, and others, but not with the consumption of antidepressants and/or anxiolytics. In the anxiety subscale, severe or very severe anxiety correlated with being in the third or fourth year of pharmacy school, the presence of reported changes in body weight, and the consumption of acetaminophen, antihistamines, NSAIDs, multivitamins, minerals, and antidepressants and/or anxiolytics. In the stress subscale, severe to very severe stress was associated with residence in Baalbek-Hermel and Bekaa, being a smoker, consuming caffeinated beverages or alcohol, the presence of weight changes, and the consumption of the same classes of medications as for anxiety, as mentioned above.

### 3.5. Results of the Multivariable Analysis

In multivariable analysis, smoking at least 10 cigarettes per day and/or at least 3 shisha per week was significantly associated with mild to moderate (aOR = 2.72, 95% CI: 1.13; 6.54, *p*-value: 0.03) and severe to extremely severe (aOR = 3.32, 95% CI: 1.30; 8.52, *p*-value: 0.01) depression, whereby our findings are suggestive of a dose-dependent association, with a more than two-fold increase in the odds of depressive symptoms for heavy smokers compared with non-smokers. Interestingly, feeling isolated from others due to COVID-19 (mild to moderate depression: aOR = 4.21, 95% CI: 1.87; 9.46, *p*-value = 0.001; severe to extremely severe depression: aOR = 11.1, 95% CI: 3.84; 32, *p*-value < 0.001) and taking other drugs (mild to moderate depression: aOR = 2.12, 95% CI: 1.06; 4.28, *p*-value = 0.04; severe to extremely severe depression: aOR = 3.93, 95% CI: 1.84; 8.38, *p*-value < 0.001) was associated with higher depression scores. Furthermore, weight gain (aOR = 2.37, 95% CI: 1.22; 4.63, *p*-value = 0.01) as well as weight loss (aOR = 3.47, 95% CI: 1.80; 6.67, *p*-value < 0.001) were significantly associated with severe to extremely severe depression during the COVID-19 pandemic.

Additionally, weight loss (aOR = 1.83, 95% CI: 1.03; 3.25, *p*-value: 0.04), being in the fifth year of pharmacy or PharmD study (aOR = 0.5, 95% CI: 0.28; 0.92, *p*-value: 0.03), taking multivitamins or minerals (aOR = 1.86, 95% CI: 1.07; 3.21, *p*-value: 0.03), and feeling isolated due to COVID-19 (aOR = 3.11, 95% CI: 1.32; 7.32, *p*-value: 0.009) had a positive significant association with having severe to extremely severe anxiety during COVID-19.

As for the stress subscale, it was noted that smoking at least 10 cigarettes per day and/or at least 3 shisha per week (aOR = 3.01, 95% CI: 1.42; 6.62, *p*-value: 0.04) and being in the third or fourth year of pharmacy study (aOR = 1.82, 95% CI: 1.10; 3.02, *p*-value: 0.02) were significantly associated with mild to moderate stress. Furthermore, body weight changes (weight loss: aOR = 3.11, 95% CI: 1.53; 6.32, *p*-value: 0.002; weight gain: aOR = 2.21, 95% CI: 1.05; 4.67, *p*-value: 0.04), taking antidepressants and/or anxiolytics (aOR = 11.16, 95% CI: 1.94; 64.4, *p*-value: 0.007), and feeling isolated due to COVID-19 (aOR = 7.10, 95% CI: 1.90; 26.6, *p*-value: 0.004) were significantly associated with severe to extremely severe stress. The details of the multivariable analysis are shown in Table 5.

In the BRS, normal to high resilience was significantly associated with first or second year in pharmacy school, being a nonsmoker, not consuming caffeinated beverages, absence of weight changes, family income below 6,000,000 Lebanese pounds, or taking antidepressants and/or anxiolytics (Table 6).

The results of the BRS revealed that almost 40% of the participants had low resilience. It was noted that being in the third or fourth year of pharmacy study (aOR = 0.63, 95% CI: 0.41; 0.97, *p*-value: 0.04), smoking at least 10 cigarettes per day and/or at least 3 shisha per week (aOR = 0.49, 95% CI: 0.26; 0.95, *p*-value: 0.03), and the consumption of caffeinated beverages (less than two cups per day: aOR = 0.54, 95% CI: 0.34; 0.85, *p*-value: 0.008; two or more cups per day: aOR = 0.56, 95% CI: 0.33; 0.96, *p*-value: 0.03) were significantly associated with low resilience. However, a high resilience was significantly associated with having a family income of more than 6,000,000 LBP per month (aOR = 1.97, 95% CI: 1.97; 3.47, *p*-value: 0.02). Table 7 shows the multivariable correlations for the BRS.

### 3.6. Results of COVID-19 Stressors

**Figure 2:**
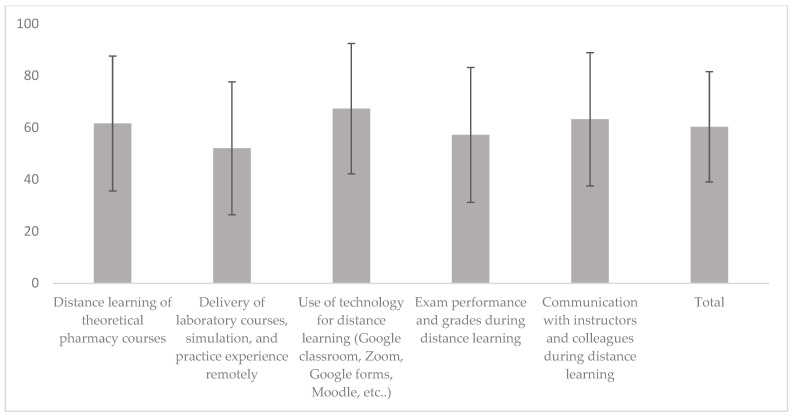
Satisfaction of participants with distance learning during COVID-19. The mean scores on the 5-point Likert scale were converted into percentages with a score of 0 corresponding to 0% and a score of 5 corresponding to 100%.

Regarding economic COVID-19 stressors, among the 127 employed participants (23%), 8% had lost employment due to COVID-19, and 41% experienced a reduction in employment due to the pandemic. Furthermore, 23% and 62% of participants’ parents lost employment or had a salary reduction due to COVID-19 (Table 2), respectively. Regarding educational stressors related to distance learning during COVID-19, the mean score of satisfaction with online learning was 60.3 ± 21.3%. The highest score (67.3 ± 25.1%) was for satisfaction with technology and online learning tools, while the lowest score (52 ± 25.6%) was for laboratory, simulation, and practice courses (Figure 2). Regarding health stressors, about 38% of the participants and 58% of their close family members had been infected with COVID-19. Feelings of isolation due to COVID-19 were variable, with 11% never feeling isolated, 22% always feeling isolated, and 45% usually feeling isolated (Table 2). Those feelings of isolation correlated with higher levels of depression, anxiety, and stress in the DASS-21 scale.

### 3.7. Vaccine Attitudes

Only 5% of the participants were vaccinated, and the Pfizer-BioNTech vaccine was the most commonly taken by those vaccinated (70%). Among the vaccinated participants, 87% trusted the vaccines’ benefits and role in controlling the pandemic, and only 37% were worried about vaccine side effects. Furthermore, 30% of those vaccinated trusted the governmental plan for the distribution and administration of COVID-19 vaccines. Among those who were not vaccinated (95%), 62% were planning to be vaccinated when they became eligible, 59% trusted vaccine benefits, and 34% were worried about vaccine side effects. However, 53% of the unvaccinated participants did not trust the governmental plan for the distribution and administration of COVID-19 vaccines. There were no significant associations between vaccine status and attitudes and any of the DASS-21 or BRS parameters.

## 4. Discussion

The current study was the first to examine the levels of depression, anxiety, and stress among Lebanese pharmacy students one year after the declaration of the COVID-19 pandemic, in addition to unveiling their resilience levels and learning processes during the pandemic. As the country continues to struggle with multiple-fold crises due to political stagnation, a deteriorating economy and currency, and corrupt governance [39], mental health issues in the time of COVID-19 in Lebanon remain largely unaddressed, especially in academia, underscoring the need to examine the pandemic’s effects on an already stressed educational system. In this baseline analysis of pharmacy students, more than 60% of the participants showed variable levels of depression, more than 70% showed variable levels of anxiety, and almost half of the participants were stressed to diverse extents. These rates of the three mental conditions are much higher than those reported during COVID-19 using the DASS-21 in a recent study [40]. Such a status of the mental health of pharmacy students at the time of the pandemic was accompanied by low resilience observed in about 40% of the participants, also lower than reported in other studies using the BRS during the pandemic [41,42]. Taken together, these data add to the available evidence on the influence of the pandemic on the mental health of students [9], including those in health-related schools [43,44], and are in context with previous studies addressing the mental health of pharmacy students in the wake of the global pandemic [27,28,29,30].

Parallel to our findings, and in addition to the health, economic, and social implications, the psychological impacts of COVID-19 are increasingly reported. According to a recent review, high burdens of depression, anxiety, stress, panic attacks, impulsivity, sleep disorders, and emotional disturbance are observed globally. Moreover, several factors associated with mental problems in COVID-19 are noticeable, including age, gender, marital status, education, occupation, income, contact with COVID-19 patients, comorbid physical and mental problems, COVID-19 related news, risk of contracting COVID-19, and others. This suggests that a psychiatric epidemic concurrent with COVID-19 may be affecting health and wellbeing [45]. After a position paper in 2020 regarding research priorities for mental health in COVID-19 especially among vulnerable groups [46], university students were tackled as a main category for whom investigating mental health liability is urgent [16]. With universities across the world having either postponed or canceled campus activities including classes, workshops, conferences, and other events in an attempt to contain the pandemic [12], online classes became a key component for the continuity of education [47]. This unexpected transition from traditional, face-to-face to online delivery has extensively impacted students’ learning experiences, due to considerable academic and everyday difficulties during the pandemic [16]. The complications associated with distance learning and the social isolation brought on by the pandemic contributed to a substantial increase in anxiety and depression among university students [9]. Moreover, student stress regarding online teaching, academic performance, the completion of each semester, the uncertainty of exam dates, and the status of the following semester, was also described [48]. With the exceptional scenario of Lebanon after October 2019, a serious social and financial deadlock overlays all of the aforementioned difficulties. Moreover, a huge blast, caused by the explosion of thousands of tons of ammonium nitrate in the Port of Beirut, the capital city, occurred on 4 August 2020, existing as an additional insult to the deteriorating status of Lebanon, and creating a tragic memory that will dwell forever in the perception of every Lebanese national. As such, and with all such calamities, an examination of students’ mental status and associated constructs after the pandemic, all economic and social changes, and the online educational shift, is required, and this appears distinctive for pharmacy students, whose mental health has been increasingly concerning to faculty and administrators, even prior to the pandemic [24,49,50].

Looking at demographic characteristics of participants, most of them were female, in parallel to enrollment trends at the School of Pharmacy, where around 70% of all enrolled students are females. This is also in line with the pharmacy workforce in Lebanon, where over 60% are female [51]. The male representation was lower, but this was unavoidable due to the lower proportion of male students among all pharmacy students. Looking into the lifestyle features of the participants, almost 14% consumed alcohol, 15% consumed energy drinks, 21% were smokers, while about 74% consumed caffeinated beverages. These percentages are somewhat in conformity with those reported in the general young population of Lebanon according to a recent investigation [52]. Whether or not these percentages are higher than the baseline percentages prior to the pandemic cannot be determined from the results of the current study, since they were not assessed in our participants prior to COVID-19. However, researchers from Jordan have pointed out higher rates of smoking, and higher consumption rates of caffeine and energy drinks in medical students performing online examinations during COVID-19 [53]. The smoking rate of 21% is higher than that reported among pharmacy students in other countries like Tunisia, with a prevalence of 19% [54], and Serbia, with a prevalence of 17% [55], while it is lower than a previously observed rate of 26% among Lebanese medical students in 2013 [56]. It is tempting to investigate whether smoking behavior in Lebanese pharmacy students is affected by online education, the country’s deteriorating status, or the fear of high COVID-19 complications and mortality in smokers [57]. Although the discussion of lifestyle and health parameters of the participants is outside the scope of this study, this is a simple snapshot of pharmacy students’ mental conditions during the combined educational shift due to the pandemic and the multiple crises of Lebanon.

The rate of mild to extremely severe depression in this study was about 64%, much higher than reported from a Spanish study on university students during COVID-19 [58]. It is also higher than the depression prevalence reported in Lebanese young adults in a study realized in May 2020, about one year prior to this study [52]. The same applies to anxiety, with a rate of about 70% according to our findings. It is possible that the cumulative effect of another year within the pandemic, the uncertain status of remote education, and the country sinking more deeply into its draining crises, may together correspond to a worsening of mental health and an increase in depression and anxiety among the surveyed students. In analyzing the associated factors of depression, the results of this study showed significant associations of depression with smoking, body weight loss, and the consumption of medications. On the other hand, weight loss, being in a senior pharmacy year (5th year or PharmD), and the consumption of multivitamins or minerals were significantly associated with higher levels of anxiety. Furthermore, feelings of isolation due to COVID-19 were significantly associated with higher levels of depression, anxiety, and stress. This finding has been previously replicated elsewhere [59], with evidence of the negative impact of social isolation on mental health, as well as on poor sleep quality and physical inactivity during confinement. As such, the mental health of pharmacy students should be addressed as isolation and remote activities continue. Perhaps this could involve a multicomponent program where psychological strategies and health counseling are recommended.

About 48% of the participants had mild to extremely severe stress. Mild to moderate stress was associated with smoking at least 10 cigarettes per day and/or at least 3 shisha per week and being in the third or fourth year of pharmacy study. On the other hand, severe to extremely severe stress was significantly associated weight gain, and more significantly with weight loss, and with the taking of antidepressants and/or anxiolytics. In pharmacy education, students’ stress is considered among the factors that may influence their professional behavior, and there is interest in monitoring it regularly. Furthermore, colleges of pharmacy have called for the development of universal stress assessment [60]. Thus, the examination of students’ stress is important to follow up their academic progress and implement preventive and restorative interventions. Given the continuously evolving status of the COVID-19 pandemic [61], these results could aid in the planning and implementation of future strategies to combat stress among pharmacy students. According to the above, specific attention should be directed to stress experienced by isolated students, smokers, those with weight fluctuation, and those taking psychoactive medications.

In the BRS, almost 40% of the participants showed low resilience, associated with being in the third or fourth year of pharmacy study, smoking, and the consumption of caffeinated beverages. Previously, low resilience in adolescents was significantly associated with the female gender, attending a private school, higher birth order compared to first born, urban residence, and physical inactivity [62]. Among health professionals, resilience was described as multifaceted, adjoining discrete personal traits in addition to social and workplace features [63]. In a survey of resilience among educators from three countries during COVID-19, internal, interpersonal, and external aspects of educational systems were needed to enhance resilience in the face of phenomenal changes like the pandemic. Additionally, support systems, strong academic leadership, trust, self-motivation, and communication were emphasized [64]. While these correlates were not clear in our population, it can be anticipated that low resilience in third year students may relate to the pressure induced by selection to professional years (as our curriculum of the Bachelor of Pharmacy program includes a pre-professional 2-year cycle followed by selection to a professional 3-year cycle), with a major upgrade in the level of courses together with the educational disruption caused by the online shift. For fourth year students, low resilience may be the pooled result of the second year in the pandemic with online learning, and the lack of face-to-face encounters and activities that pharmacy students normally have in the third and fourth years of study. While these explanations remain preliminary and cannot be authenticated within the framework of this study, and while some associated factors remain unmodifiable, these findings can be used to guide the identification of pharmacy students who need greater support during perplexing transitions such as those accompanying the pandemic. High resilience among the participants was significantly associated with having a family income of more than 6,000,000 LBP per month. Cosco and Colleagues [65] have previously reported that higher social classes had higher resilience compared to lower classes. With Lebanon’s deteriorating economy and monetary inflation, it is likely that a better income and a sense of relative financial security supports better mental states, including higher resilience. Research with medical, nursing, and pharmacy students demonstrates correlations of resilience with positive well-being and academic success [66]. Likewise, in a study of the general public in Poland that used the BRS, enhancing resilience was found to reduce negative mental effects during the pandemic [67]. As such, the development of resilience in our students will better prepare them for academic achievement and ensure a smooth transition into their professional roles, especially amidst various educational, financial, and health challenges.

Stressors that can affect students were assessed in this study, where the loss of employment or reduction of monetary income for the formerly employed students or their family members was noticeable. Although this was reported in earlier COVID-19 studies on university students [9], its impact needs to be emphasized in a country facing a deteriorating economy and financial collapse. Similarly, educational stressors resulting from the shift to remote education were assessed, and although overall satisfaction was about 60% and students were satisfied with online tools, technological platforms, and communication, decreased satisfaction was also conspicuous. This was connected to delivery of material with a typical practice component, like laboratories, simulation courses, and experiential education. For pharmacy educators and students alike, the pandemic has been like no other time in academia. In the course of several weeks, pharmacy education underwent more significant changes than it arguably had experienced in the previous decade. The changes were often made on a daily basis as the depth and breadth of the problems caused by the pandemic were apprehended. These problems challenged the established educational processes, and disrupted the fundamental elements of community, time, and place that form foundations for the success of pharmacy education. In an analysis of distance learning during COVID-19 [68], it was considered to be a complex field with separate understandings and limited attention; nevertheless, it demonstrated resilience in higher education, with the ability to react and to re-organize itself within a short time, perhaps creating a stronger university, able to combine quality in education with potential technological advances. As much as these changes may have been loaded with new opportunities for learning, they also have been overwhelming to students, and have added to their stressful encounters. Regarding health stressors, COVID-19 and feelings of isolation due to the infection were clear. In a study from Turkey, it was observed that the danger of infection was the highest stressor among university students assessed during the pandemic [69]. Although our study did not rate the economic, educational and health stressors for students, feelings of isolation due to the pandemic correlated with higher levels of depression, anxiety, and stress in the DASS-21 scale, making that a major health stressor that needs to be addressed, should mental health support be given to students.

At the time of the administration of this survey, the vaccination plan in Lebanon was in the early stages of implementation, and only 5% of the participants were vaccinated. However, the majority of vaccinated students (87%) and those who were not yet vaccinated (59%) trusted the vaccine. There was a noticeable difference in trusting the national vaccination plan among those vaccinated (trusted by 30%) and those not vaccinated (trusted by 47%). Our study did not detect significant associations between vaccine status and attitudes and any of the DASS-21 or BRS parameters. In fact, the expedited development and relative novelty of the COVID-19 vaccines have led to public uncertainty, and efforts to explain the action of these vaccines involve a degree of complexity in immune responses and genetic mechanisms, which are difficult to communicate quickly and simply [70]. Nevertheless, with our students having the scientific background of a health-related major, it is possible that their knowledge might have reduced their worries about vaccines, thereby avoiding a detectable significance of vaccine attitudes on their mental health. This, however, needs further investigation.

This study does have limitations. The population belonged to a single school of pharmacy; with the variety of educational cultures across the five pharmacy schools in Lebanon, a more inclusive study would be more agile in revealing mental health and resilience over a larger, more representative population. Moreover, and since no formal investigation of the mental health of pharmacy students was purposefully conducted prior to the pandemic, it was not feasible to use a baseline reference to check particular changes in mental health and resilience after COVID-19. As a descriptive, cross-sectional study, the findings cannot be used to estimate the incidence of depression, anxiety, and stress, nor to generalize the mental status of participants over a long period of time, but rather portrays this status at the time of survey administration. Furthermore, the recall bias and nonresponse bias of this study design cannot be eliminated.

The implications of the current study in evaluating mental health and resilience in pharmacy students may contribute to the current as well as future research on global threats such as pandemics. As higher education institutions discuss future educational strategies in light of the pandemic, the available constructs and factors which affect depression, anxiety, stress, and resilience among pharmacy students could help to reshape the architecture of pharmacy education in a way that considers mental health within ongoing processes of improvement. This includes a straightforward appreciation of the study population, pharmacy students, where rigorous, focused, and multifaceted approaches to improve their mental health and increase their resilience should never be overlooked.

## 5. Conclusions

In conclusion, this exploratory investigation addressed the mental health and resilience of pharmacy students in Lebanon one year after the declaration of COVID-19. The mental health and resilience of pharmacy students in this study were assessed within the collateral shadows of the pandemic, with the added effects of online education about one year after the pandemic’s onset. This preliminary analysis revealed that depression, anxiety, and stress were detected, while resilience was low. These observations, associated with the COVID-19 pandemic after almost one year, have been related to various demographic, lifestyle, and educational factors. The mental traits of pharmacy students amidst the pandemic have been incorporated in the social, academic, and physical burdens of the university years, which are pivotal in terms of mental development and psychological health. Despite the prelusive, observational design of this study, it is essential to consider the implications of this and similar studies on pharmacy education. As the world slowly recovers from COVID-19, it is essential to ascertain the long-term mental effects of the pandemic and take the necessary precautions to support the mental health of today’s university students.

## Figures and Tables

**Table 1:** Sociodemographic characteristics of participants and reported lifestyle changes in relation to COVID-19.

		*n* (%)
**Gender**	Female	428 (76.3)
**Age; Median (Q1;Q3)**		22 (20; 23)
**Min–Max**		17–42
**Nationality**	Lebanese	532 (94.8)
	Non-Lebanese	29 (5.2)
**Area of residence**	Beirut	131 (23.4)
	Baalbek-Hermel and Bekaa	144 (25.7)
	Mount Lebanon	118 (21)
	Nabatieh and South	81 (14.4)
	North and Akkar	80 (14.3)
	Residing currently out of Lebanon	7 (1.2)
**Marital status**	Single	524 (93.4)
	Married	35 (6.2)
	Widowed/Divorced	2 (0.4)
**Household size**	One to two persons	22 (3.9)
	Three to five	351 (62.6)
	Six or more	188 (33.5)
**Year in pharmacy school**	First or second	174 (31)
	Third or fourth	219 (39)
	Fifth or PharmD	168 (29.9)
**Smoking**	No	445 (79.3)
	<10 cigarettes per day and/or <3 shisha per week	66 (11.8)
	≥10 cigarettes per day and/or ≥3 shisha per week	50 (8.9)
**Consuming caffeinated beverages**	No	148 (26.4)
	Less than two cups per day	254 (45.4)
	Two cups or more per day	158 (28.2)
**Consuming energy drinks**	No	477 (85)
	Once per week	61 (10.9)
	More than once per week	23 (4.1)
**Drinking alcohol during COVID-19**	No	484 (86.3)
	Once every few weeks On a weekly basis	59 (10.5) 18 (3.2)
**Body weight changes during COVID-19**	No changes	161 (28.7)
	Gained weight	181 (32.3)
	Lost weight	219 (39)
**Family income per month in LBP**	<2,000,000	126 (22.5)
	[2,000,000–4,000,000]	199 (35.5)
	[4,000,001–6,000,000]	123 (21.9)
	>6,000,000	113 (20.1)
**Family income affected by lockdown due to COVID-19**	Yes	407 (72.5)
**Receiving financial support in foreign currency from family/relatives living outside Lebanon**	Yes	97 (17.3)

COVID-19, Coronavirus disease 2019; LBP, Lebanese pounds; Q1, first quartile; Q3, third quartile.

**Table 2:** Health- and COVID-19-related information of the participants.

		*n* (%)
**Medication taken during COVID-19**		
**Acetaminophen**	Yes	118 (21)
**Antihistamines**	Yes	72 (12.8)
**NSAIDs**	Yes	84 (15)
**Multivitamins or minerals**	Yes	168 (29.9)
**Antidepressants and/or anxiolytics**	Yes	17 (3)
**Others**	Yes	72 (12.8)
**Being employed**	Yes	127 (22.6)
**Lost employment during COVID-19**	Yes	10 (7.9)
**Employment reduction during COVID-19**	Yes	51 (41.2)
**Parents lost employment during COVID-19**	Yes	128 (22.8)
**Parents experienced reduction in salary during COVID-19**	Yes	346 (61.7)
**Being diagnosed with COVID-19**	No	346 (61.7)
	Yes with mild to moderate symptoms	157 (28)
	Yes with severe symptoms or hospitalization	58 (10.3)
**Close family diagnosed with COVID-19**	No	236 (42.1)
	Yes with mild to moderate symptoms	169 (30.1)
	Yes with severe symptoms or hospitalization	156 (27.8)
**Feel isolated due to COVID-19**	Never	61 (10.9)
	Rarely	123 (21.9)
	Usually	255 (45.5)
	Always	122 (21.7)
**Received any COVID-19 vaccine**	Yes	30 (5.3)
**Vaccine type**	Sputnik V	5 (16.7)
	Sinopharm	3 (10)
	Pfizer	21 (70)
	AstraZeneca	1 (3.3)
**Trust COVID-19 vaccine benefits and the role of vaccines in controlling the pandemic**	No	0
	Yes	26 (86.7)
	Somewhat	4 (13.7)
**Worried about the side effects of COVID-19 vaccines**	No	13 (43.3)
	Yes	11 (36.7)
	Somewhat	6 (20)
**Trust the governmental plan for the distribution and administration of COVID-19 vaccines**	No	13 (43.3)
	Yes	9 (30)
	Somewhat	8 (26.7)
**Received any COVID-19 vaccine**	No	531 (94.7)
**Plan to be vaccinated when eligible for COVID-19 vaccines**	No	76 (14.3)
	Yes	330 (62.1)
	Maybe	125 (23.5)
**Trust COVID-19 vaccine benefits and the role of vaccines in controlling the pandemic**	No	61 (11.6)
	Yes	310 (59)
	Somewhat	154 (29.3)
**Worried about the side effects of COVID-19 vaccines**	No	161 (30.4)
	Yes	183 (34.5)
	Somewhat	186 (35.1)
**Trust the governmental plan for the distribution and administration of COVID-19 vaccines**	No	278 (52.5)
	Yes	92 (17.4)
	Somewhat	160 (30.2)

COVID-19, Coronavirus disease 2019; NSAIDs, Non-Steroidal Anti-Inflammatory Drugs.

**Table 3:** Results of DASS-21 and BRS analysis of the participants.

Scale	Result	*n* (%)
DASS-21		
*Depression subscale*	Normal	202 (36)
	Mild to moderate	210 (37.4)
	Severe to extremely severe	149 (26.6)
*Anxiety subscale*	Normal	164 (29.2)
	Mild to moderate	191 (34)
	Severe to extremely severe	206 (36.7)
*Stress subscale*	Normal	290 (51.7)
	Mild to moderate	172 (30.7)
	Severe to extremely severe	99 (17.6)
BRS scale	Low	221 (39.4)
	Normal to high	340 (60.6)

DASS-21, Depression, Anxiety, and Stress Scale; BRS, Brief Resilience Scale.

**Table 4:** Bivariate associations for DASS-21 subscales.

	Depression Subscale			Anxiety Subscale			Stress Subscale		
	Normal *n* = 202	Mild to Moderate *n* = 210	Severe to Extremely Severe *n* = 149	Normal *n* = 164	Mild to Moderate *n* = 191	Severe to Very Severe *n* = 206	Normal *n* = 290	Mild to Moderate *n* = 172	Severe to Very Severe *n* = 99
**Gender**									
Male	52 (39.1	45 (33.8)	36 (27.1)	47 (35.3)	43 (32.3)	43 (32.3)	71 (53.4)	41 (30.8)	21 (15.8)
Female	150 (35)	165 (38.6)	113 (26.4)	117 (27.3)	148 (34.6)	163 (38.1)	219 (51.2)	131 (30.6)	78 (18.2)
**Age**	202 (22.3 ± 3.6)	210 (21.8 ± 2.6)	148 (21.7 ± 2.6)	164 (22.3 ± 3.8)	191 (22.0 ± 2.8)	205 (21.8 ± 2.5)	289 (22 ± 3.4)	172 (22 ± 2.3)	99 (21.6 ± 2.9)
**Nationality**									
Lebanese	192 (36.1)	198 (37.2)	142 (26.7)	153 (28.8)	181 (34)	198 (37.2)	273 (51.3)	167 (31.4)	92 (17.3)
Non-Lebanese	10 (34.5)	12 (41.4)	7 (24.1)	11 (37.9)	10 (34.5)	8 (27.6)	17 (58.6)	5 (17.2)	7 (24.1)
**Area of residence**									
Beirut	48 (36.6)	47 (35.9)	36 (27.5)	36 (27.5)	43 (32.8)	52 (39.7)	67 (51.1)	40 (30.5)	24 (18.3) *
Baalbek-Hermel and Bekaa	46 (31.9)	51 (35.4)	47 (32.6)	33 (22.9)	47 (32.6)	64 (44.4)	66 (45.8)	42 (29.2)	36 (25)
Mount Lebanon	49 (41.5)	41 (34.7)	28 (23.7)	40 (33.9)	48 (40.7)	30 (25.4)	72 (61)	27 (22.9)	19 (16.1)
Nabatieh and South	27 (33.3)	40 (49.4)	14 (17.3)	25 (30.9)	32 (39.5)	24 (29.6)	38 (46.9)	31 (38.3)	12 (14.8)
North and Akkar	30 (37.5)	30 (37.5)	20 (25)	28 (35)	20 (25)	32 (40)	45 (56.3)	27 (33.8)	8 (10)
Currently out of Lebanon	2 (28.6)	1 (14.3)	4 (57.1)	2 (28.6)	1 (14.3)	4 (57.1)	2 (28.6)	5 (71.4)	0
**Marital status**									
Single	188 (35.9)	197 (37.6)	139 (26.5)	154 (29.4)	180 (34.4)	190 (36.3)	268 (51.1)	161 (30.7)	95 (18.1)
Married	13 (37.1)	13 (37.1)	9 (25.7)	10 (28.6)	10 (28.6)	15 (42.9)	21 (60)	11 (31.4)	3 (8.6)
Widow/Divorced	1 (50)	0	1 (50)	0	1 (50)	1 (50)	1 (50)	0	1 (50)
**Household size**									
One to two persons	6 (27.3)	4 (18.2)	12 (54.5) *	6 (27.3)	5 (22.7)	11 (50)	11 (50)	6 (27.3)	5 (22.7)
Three to five	123 (35)	136 (38.7)	92 (26.2)	104 (29.6)	118 (33.6)	129 (36.8)	184 (52.4)	105 (29.9)	62 (17.7)
Six or more	73 (38.8)	70 (37.2)	45 (23.9)	54 (28.7)	68 (36.2)	66 (35.1)	95 (50.5)	61 (32.4)	32 (17)
**Year in pharmacy school**									
First or second	63 (36.2)	67 (38.5)	44 (25.3)	56 (32.2)	61 (35.1)	57 (32.8) **	100 (57.5)	43 (24.7)	31 (17.8) **
Third or fourth	73 (33.3)	83 (37.9)	63 (28.8)	46 (21)	78 (35.6)	95 (43.4)	92 (42)	83 (37.9)	44 (20.1)
Fifth or PharmD	66 (39.3)	60 (35.7)	42 (25)	62 (36.9)	52 (31)	54 (32.1)	98 (58.3)	46 (27.4)	24 (14.3)
**Smoking**									
No	170 (38.2)	165 (37.1)	110 (24.7) *	133 (29.9)	155 (34.8)	157 (35.3)	244 (54.8)	125 (28.1)	76 (17.1) **
<10 cigarettes per day and/or <3 shisha per week	23 (34.8)	24 (36.4)	19 (28.8)	21 (31.8)	19 (28.8)	26 (39.4)	33 (50)	23 (34.8)	10 (15.2)
≥10 cigarettes per day and/or ≥3 shisha per week	9 (18)	21 (42)	20 (40)	10 (20)	17 (34)	23 (46)	13 (26)	24 (48)	13 (26)
**Consuming caffeinated beverages**									
No	57 (38.5)	60 (40.5)	31 (20.9)	48 (32.4)	53 (35.8)	47 (31.8) *	83 (56.1)	44 (29.7)	21 (14.2) *
Less than two cups per day	100 (39.4)	88 (34.6)	66 (26)	78 (30.7)	93 (36.6)	83 (32.7)	138 (54.3)	78 (30.7)	38 (15)
Two cups or more per day	45 (28.5)	61 (38.6)	52 (32.9)	38 (24.1)	44 (27.8)	76 (48.1)	69 (43.7)	49 (31)	40 (25.3)
**Consuming energy drinks**									
No	178 (37.3)	176 (36.9)	123 (25.8)	144 (30.2)	161 (33.8)	172 (36.1)	252 (52.8)	139 (29.1)	86 (18)
Once per week	19 (31.1)	25 (41)	17 (27.9)	17 (27.9)	22 (36.1)	22 (36.1)	31 (50.8)	22 (36.1)	8 (13.1)
More than once per week	5 (21.7)	9 (39.1)	9 (39.1)	3 (13)	8 (34.8)	12 (52.2)	7 (30.4)	11 (47.8)	5 (21.7)
**Drinking alcohol during COVID-19**									
No	180 (37.2)	189 (39)	115 (23.8) **	141 (29.1)	169 (34.9)	174 (36)	262 (54.1)	142 (29.3)	80 (16.5) *
Once every few weeks	14 (23.7)	17 (28.8)	28 (47.5)	16 (27.1)	18 (30.5)	25 (42.4)	20 (33.9)	23 (39)	16 (27.1)
On a weekly basis	8 (44.4)	4 (22.2)	6 (33.3)	7 (38.9)	4 (22.2)	7 (38.9)	8 (44.4)	7 (38.9)	3 (16.7)
**Body weight changes during COVID-19**									
No changes	78 (48.5)	62 (38.5)	21 (13) ***	60 (37.3)	56 (34.8)	45 (28) *	105 (65.2)	41 (25.5)	15 (9.3) **
Gained weight	65 (35.9)	56 (30.9)	60 (33.1)	49 (27.1)	56 (30.9)	76 (42)	87 (48.1)	57 (31.5)	37 (20.4)
Lost weight	59 (26.9)	92 (42)	68 (31.1)	55 (25.1)	79 (36.1)	85 (38.8)	98 (44.7)	74 (33.8)	47 (21.5)
**Family income per month in LBP**									
<2,000,000	37 (29.4)	56 (44.4)	33 (26.2)	32 (25.4)	39 (31)	55 (43.7)	60 (47.6)	44 (34.9)	22 (17.5)
[2,000,000–4,000,000]	74 (37.2)	64 (32.2)	61 (30.7)	52 (26.1)	74 (37.2)	73 (36.7)	102 (51.3)	58 (29.1)	39 (19.6)
[4,000,001–6,000,000]	43 (35)	54 (43.9)	26 (21.1)	41 (33.3)	40 (32.5)	42 (34.1)	65 (52.8)	43 (35)	15 (12.2)
>6,000,000	48 (42.5)	36 (31.9)	29 (25.7)	39 (34.5)	38 (33.6)	36 (31.9)	63 (55.8)	27 (23.9)	23 (20.4)
**Family income affected by lockdown due to COVID-19**									
No	58 (37.7)	64 (41.6)	32 (20.8)	48 (31.2)	58 (37.7)	48 (31.2)	78 (50.6)	55 (35.7)	21 (13.6)
Yes	144 (35.4)	146 (35.9)	117 (28.7)	116 (28.5)	133 (32.7)	158 (38.8)	212 (52.1)	117 (28.7)	78 (19.2)
**Receiving financial support in foreign currency from family/relatives living outside Lebanon**									
No	171 (36.9)	170 (36.6)	123 (26.5)	136 (29.3)	160 (34.5)	168 (36.2)	237 (51.1)	149 (32.1)	78 (16.8)
Yes	31 (32)	40 (41.2)	26 (26.8)	28 (28.9)	31 (32)	38 (39.2)	53 (54.6)	23 (23.7)	21 (21.6)
**Taking acetaminophen during COVID-19**									
No	171(38.6)	164 (37)	108 (24.4) *	142 (32.1)	157 (35.4)	144 (32.5) ***	243 (54.9)	133 (30)	67 (15.1) **
Yes	31 (26.3)	46 (39)	41 (34.7)	22 (18.6)	34 (28.8)	62 (52.5)	47 (39.8)	39 (33.1)	32 (27.1)
**Taking antihistamines during COVID-19**									
No	184 (37.6)	183 (37.4)	122 (24.9) *	153 (31.3)	172 (35.2)	164 (33.5) ***	264 (54)	150 (30.7)	75 (15.3) ***
Yes	18 (25)	27 (37.5)	27 (37.5)	11 (15.3)	19 (26.4)	42 (58.3)	26 (36.1)	22 (30.6)	24 (33.3)
**Taking NSAIDs during COVID-19**									
No	184 (38.6)	180 (37.7)	113 (23.7) ***	155 (32.5)	163 (34.2)	159 (33.3) ***	259 (54.3)	148 (31)	70 (14.7) ***
Yes	18 (21.4)	30 (35.7)	36 (42.9)	9 (10.7)	28 (33.3)	47 (56)	31 (36.9)	24 (28.6)	29 (34.5)
**Taking multivitamins or minerals during COVID-19**									
No	153 (38.9)	147 (37.4)	93 (23.7) *	133 (33.8)	143 (36.4)	117 (29.8) ***	220 (56)	120 (30.5)	53 (13.5) ***
Yes	49 (29.2)	63 (37.5)	56 (33.3)	31 (18.5)	48 (28.6)	89 (53)	70 (41.7)	52 (31)	46 (27.4)
**Taking antidepressants and/or anxiolytics during COVID-19**									
No	199 (36.6)	204 (37.5)	141 (25.9)	163 (30)	186 (34.2)	195 (35.8) *	288 (52.9)	166 (30.5)	90 (16.5) ***
Yes	3 (17.6)	6 (35.3)	8 (47.1)	1 (5.9)	5 (29.4)	11 (64.7)	2 (11.8)	6 (35.3)	9 (52.9)
**Taking other drugs during COVID-19**									
No	185 (37.8)	182 (37.2)	122 (24.9) *	149 (30.5)	166 (33.9)	174 (35.6)	262 (53.6)	145 (29.7)	82 (16.8)
Yes	17 (23.6)	28 (38.9)	27 (37.5)	15 (20.8)	25 (34.7)	32 (44.4)	28 (38.9)	27 (37.5)	17 (23.6)
**Being employed**									
No	155 (35.7)	159 (36.6)	120 (27.6)	124 (28.6)	147 (33.9)	163 (37.6)	223 (51.4)	128 (29.5)	83 (19.1)
Yes	47 (37)	51 (40.2)	29 (22.8)	40 (31.5)	44 (34.6)	43 (33.9)	67 (52.8)	44 (34.6)	16 (12.6)
**Parents lost employment during COVID-19**									
No	171 (39.5)	150 (34.6)	112 (25.9) **	139 (32.1)	147 (33.9)	147 (33.9) **	228 (52.7)	128 (29.6)	77 (17.8)
Yes	31 (24.2)	60 (46.9)	37 (28.9)	25 (19.5)	44 (34.4)	59 (46.1)	62 (48.4)	44 (34.4)	22 (17.2)
**Parents experienced reduction in salary during COVID-19**									
No	93 (43.3)	72 (33.5)	50 (23.3) *	74 (34.4)	75 (34.9)	66 (30.7) *	126 (58.6)	58 (27)	31 (14.4) *
Yes	109 (31.5)	138 (39.9)	99 (28.6)	90 (26)	116 (33.5)	140 (40.5)	164 (47.4)	114 (32.9)	68 (19.7)
**Being diagnosed with COVID-19**									
No	140 (40.5)	114 (32.9)	92 (26.6) *	115 (33.2)	118 (34.1)	113 (32.7) ***	185 (53.5)	103 (29.8)	58 (16.8)
Yes, mild to moderate symptoms	45 (28.7)	75 (47.8)	37 (23.6)	36 (22.9)	63 (40.1)	58 (36.9)	81 (51.6)	53 (33.8)	23 (14.6)
Yes, severe symptoms or hospitalization	17 (29.3)	21 (36.2)	20 (34.5)	13 (22.4)	10 (17.2)	35 (60.3)	24 (41.4)	16 (27.6)	18 (31)
**Close family diagnosed with COVID-19**									
No	94 (39.8)	80 (33.9)	62 (26.3) **	84 (35.6)	83 (35.2)	69 (29.2) **	124 (52.5)	71 (30.1)	41 (17.4)
Yes, mild to moderate symptoms	72 (42.6)	55 (32.5)	42 (24.9)	52 (30.8)	54 (32)	63 (37.3)	93 (55)	47 (27.8)	29 (17.2)
Yes, severe symptoms or hospitalization	36 (23.1)	75 (48.1)	45 (28.8)	28 (17.9)	54 (34.6)	74 (47.4)	73 (46.8)	54 (34.6)	29 (18.6)
**Feel isolated due to COVID-19**									
Never	38 (62.3)	17 (27.9)	6 (9.8) ***	30 (49.2)	17 (27.9)	14 (23) ***	45 (73.8)	13 (21.3)	3 (4.9) ***
Rarely	58 (47.2)	51 (41.5)	14 (11.4)	44 (35.8)	50 (40.7)	29 (23.6)	75 (61)	39 (31.7)	9 (7.3)
Usually	81 (31.8)	96 (37.6)	78 (30.6)	62 (24.3)	90 (35.3)	103 (40.4)	118 (46.3)	85 (33.3)	52 (20.4)
Always	25 (20.5)	46 (37.7)	51 (41.8)	28 (23)	34 (27.9)	60 (49.2)	52 (42.6)	35 (28.7)	35 (28.7)
**Received any COVID-19 vaccine**									
No	192 (36.2)	197 (37.1)	142 (26.7)	156 (29.4)	177 (33.3)	198 (37.3)	270 (50.8)	165 (31.1)	96 (18.1)
Yes	10 (33.3)	13 (43.3)	7 (23.3)	8 (26.7)	14 (46.7)	8 (26.7)	20 (66.7)	7 (23.3)	3 (10)

For the bivariate analysis: *p* < 0.05 *, *p* < 0.01 **, *p* < 0.001 ***. COVID-19, Coronavirus disease 2019; NSAIDs, Non-Steroidal Anti-Inflammatory Drugs; LBP, Lebanese pounds.

**Table 5:** Multivariable associations for DASS-21 subscales.

		Adjusted OR (95% CI)	*p*-Value
Multivariable Associations for the Depression Subscale—*n* = 561			
***Mild to moderate*** **vs. *Normal***			
**Smoking**	No	Reference	
	<10 cigarettes per day and/or <3 shisha per week	1.14 (0.58; 2.25)	0.7
	≥10 cigarettes per day and/or ≥3 shisha per week	2.72 (1.13; 6.54)	**0.03**
**Feel isolated from others due to COVID-19**	Never	Reference	
	Rarely	1.67 (0.80; 3.50)	0.2
	Usually	2.47 (1.23; 4.95)	**0.01**
	Always	4.21 (1.87; 9.46)	**0.001**
**Taking other drugs ^#^**		2.12 (1.06; 4.28)	**0.04**
***Severe to extremely severe*** **vs. *Normal***			
**Smoking**	No	Reference	
	<10 cigarettes per day and/or <3 shisha per week	1.009 (0.46; 2.22)	0.9
	≥10 cigarettes per day and/or ≥3 shisha per week	3.32 (1.30; 8.52)	**0.01**
**Body weight during COVID-19**	No changes	Reference	
	Gained weight	2.37 (1.22; 4.63)	**0.01**
	Lost weight	3.47 (1.80; 6.67)	**<0.001**
**Taking other drugs ^#^**		3.93 (1.84; 8.38)	**<0.001**
**Feel isolated due to COVID-19**	Never	Reference	
	Rarely	1.009 (0.33; 3.08)	0.9
	Usually	5.32 (1.99; 14.2)	**0.001**
	Always	11.1 (3.84; 32)	**<0.001**
**Multivariable associations for the anxiety subscale—*n* = 561**			
***Mild to moderate*** **vs. *Normal***			
**Feel isolated due to COVID-19**	Never	Reference	
	Rarely	1.70 (0.80; 3.62)	0.2
	Usually	2.01 (1.01; 4.23)	**0.047**
	Always	1.76 (0.77; 4.01)	0.2
***Severe to extremely severe*** **vs. *Normal***			
**Body weight during COVID-19**	No changes	Reference	
	Gained weight	1.55 (0.86; 2.80)	0.1
	Lost weight	1.83 (1.03; 3.25)	**0.04**
**Year in pharmacy school**	First or second	Reference	
	Third or fourth	1.30 (0.73; 2.30)	0.4
	Fifth or PharmD	0.5 (0.28; 0.92)	**0.03**
**Taking multivitamins or minerals**		1.86 (1.07; 3.21)	**0.03**
**Feel isolated due to COVID-19**	Never	Reference	
	Rarely	1.20 (0.51; 2.82)	0.7
	Usually	2.65 (1.21; 5.79)	**0.02**
	Always	3.11 (1.32; 7.32)	**0.009**
**Multivariable associations for the stress subscale—*n* = 561**			
***Mild to moderate*** **vs. *Normal***			
**Smoking**	No	Reference	
	<10 cigarettes per day and/or <3 shisha per week	1.16 (0.60; 2.25)	0.7
	≥10 cigarettes per day and/or ≥3 shisha per week	3.01 (1.42; 6.62)	**0.004**
**Body weight during COVID-19**	No changes	Reference	
	Gained weight	1.55 (0.90; 2.64)	0.1
	Lost weight	1.85 (1.12; 3.08)	**0.02**
**Year in pharmacy school**	First or second	Reference	
	Third or fourth	1.82 (1.10; 3.02)	**0.02**
	Fifth or PharmD	0.88 (0.5; 1.54)	0.7
**Feel isolated due to COVID-19**	Never	Reference	
	Rarely	1.61 (0.74; 3.51)	0.2
	Usually	2.28 (1.10; 4.71)	**0.03**
	Always	2.09 (0.93; 4.69)	0.07
***Severe to extremely severe*** **vs. *Normal***			
**Body weight during COVID-19**	No changes	Reference	
	Gained weight	2.21 (1.05; 4.67)	**0.04**
	Lost weight	3.11 (1.53; 6.32)	**0.002**
**Taking antidepressants and/or anxiolytics**		11.16 (1.94; 64.4)	**0.007**
**Feel isolated due to COVID-19**	Never	Reference	
	Rarely	1.27 (0.30; 5.35)	0.7
	Usually	4.81 (1.34; 17.3)	**0.02**
	Always	7.10 (1.90; 26.6)	**0.004**

^#^ Others = other than acetaminophen, antihistamines, NSAIDs, multivitamins or minerals, antidepressants or anxiolytics; COVID-19, Coronavirus disease 2019; NSAIDs, Non-Steroidal Anti-Inflammatory Drugs; CI, Confidence interval.

**Table 6:** Bivariate associations for BRS.

		Low *n* = 221	Normal to High *n* = 340
**Gender**	Male	49 (36.8)	84 (63.2)
	Female	172 (40.2)	256 (59.8)
**Age**		21.8 ± 2.52	22.1 ± 3.3
**Nationality**	Lebanese	212 (39.8)	320 (60.2)
	Non-Lebanese	9 (31)	20 (69)
**Area of residence**	Beirut	47 (35.9)	84 (64.1)
	Baalbek-Hermel and Bekaa	68 (47.2)	76 (52.8)
	Mount Lebanon	43 (36.4)	75 (63.6)
	Nabatieh and South	28 (34.6)	53 (65.4)
	North and Akkar	30 (37.5)	50 (62.5)
	Currently out of Lebanon	5 (71.4)	2 (28.6)
**Marital status**	Single	213 (40.6)	311 (59.4)
	Married	8 (22.9)	27 (77.1)
	Widow/Divorced	0	2 (100)
**Household size**	One to two persons	6 (27.3)	16 (72.7)
	Three to five	139 (39.6)	212 (60.4)
	Six or more	76 (40.4)	112 (59.6)
**Year in pharmacy school**	First or second	58 (33.3)	116 (66.7) *
	Third or fourth	102 (46.6)	117 (53.4)
	Fifth or PharmD	61 (36.3)	107 (63.7)
**Smoking**	No	167 (37.5)	278 (62.5) *
	<10 cigarettes per day and/or <3 shisha per week	26 (39.4)	40 (60.6)
	≥10 cigarettes per day and/or ≥3 shisha per week	28 (56)	22 (44)
**Consuming caffeinated beverage**	No	44 (29.7)	104 (70.3) *
	Less than two cups per day Two cups or more per day	107 (42.1) 70 (44.3)	147 (57.9) 88 (55.7)
**Consuming energy drinks**	No	185 (38.8)	292 (61.2)
	Once per week	25 (41)	36 (59)
	More than once per week	11 (47.8)	12 (52.2)
**Drinking alcohol during COVID-19**	No	183 (37.8)	301 (62.2)
	Once every few weeks On a weekly basis	27 (45.8) 11 (61.1)	32 (54.2) 7 (38.9)
**Body weight changes during COVID-19**	No	50 (31.1)	111 (68.9) *
	Gained weight	78 (43.1)	103 (56.9)
	Lost weight	93 (42.5)	126 (57.5)
**Family income per month in LBP**	<2,000,000	57 (45.2)	69 (54.8) *
	[2,000,000–4,000,000]	86 (43.2)	113 (56.8)
	[4,000,001–6,000,000]	45 (36.6)	78 (63.4)
	>6,000,000	33 (29.2)	80 (70.8)
**Family income affected by lockdown due to COVID-19**	No	60 (39)	94 (61)
	Yes	161 (39.6)	246 (60.4)
**Receiving financial support in foreign currency from family/relatives living outside Lebanon**	No	187 (40.3)	277 (59.7)
	Yes	34 (35.1)	63 (64.9)
**Taking acetaminophen**	No	167 (37.7)	276 (62.3)
	Yes	54 (45.8)	64 (54.2)
**Taking antihistamines**	No	187 (38.2)	302 (61.8)
	Yes	34 (47.2)	38 (52.8)
**Taking NSAIDs**	No	180 (37.7)	297 (62.3)
	Yes	41 (48.8)	43 (51.2)
**Taking multivitamins or minerals**	No	143 (36.4)	250 (63.6) *
	Yes	78 (46.4)	90 (53.6)
**Taking antidepressants and/or anxiolytics**	No	209 (38.4)	335 (61.6) **
	Yes	12 (70.6)	5 (29.4)
**Taking other drugs**	No	186 (38)	303 (62)
	Yes	35 (48.6)	37 (51.4)
**Being employed**	No	177 (40.8)	257 (59.2)
	Yes	44 (34.6)	83 (65.4)
**Parents lost employment during COVID-19**	No	168 (38.8)	265 (61.2)
	Yes	53 (41.4)	75 (58.6)
**Parents experienced reduction in salary during COVID-19**	No	77 (35.8)	138 (64.2)
	Yes	144 (41.6)	202 (58.4)
**Being diagnosed with COVID-19**	No	141 (40.8)	205 (59.2)
	Yes, mild to moderate symptoms	56 (35.7)	101 (64.3)
	Yes, severe symptoms or hospitalization	24 (41.4)	34 (58.6)
**Close family diagnosed with COVID-19**	No	95 (40.3)	141 (59.7)
	Yes, mild to moderate symptoms	61 (36.1)	108 (63.9)
	Yes, severe symptoms or hospitalization	65 (41.7)	91 (58.3)
**Feel isolated due to COVID-19**	Never	22 (36.1)	39 (63.9) **
	Rarely	37 (30.1)	86 (69.9)
	Usually	99 (38.8)	156 (61.2)
	Always	63 (51.6)	59 (48.4)
**Received any COVID-19 vaccine**	No	209 (39.4)	322 (60.6)
	Yes	12 (40)	18 (60)

*p* < 0.05 *, *p* < 0.01 ** COVID-19, Coronavirus disease 2019; NSAIDs, Non-Steroidal Anti-Inflammatory Drugs; LBP, Lebanese pounds.

**Table 7:** Multivariable associations for BRS.

*Normal to High (Low: Reference Group)*		Adjusted OR (95% CI)	*p*-Value
**Year in pharmacy school**	First or second	Reference	
	Third or fourth	0.63 (0.41; 0.97)	0.04
	Fifth or PharmD	0.99 (0.62; 1.59)	0.9
**Smoking**	No	Reference	
	<10 cigarettes per day and/or <3 shisha per week	1.10 (0.61; 1.97)	0.7
	≥10 cigarettes per day and/or ≥3 shisha per week	0.49 (0.26; 0.95)	**0.03**
**Consuming caffeinated beverage**	No	Reference	
	Less than two cups per day Two cups or more per day	0.54 (0.34; 0.85) 0.56 (0.33; 0.96)	**0.008** **0.03**
**Family income per month in LBP**	<2,000,000	Reference	
	[2,000,000–4,000,000]	0.97 (0.60; 1.56)	0.9
	[4,000,001–6,000,000]	1.21 (0.71; 2.08)	0.5
	>6,000,000	1.97 (1.12; 3.47)	**0.02**

COVID-19, Coronavirus disease 2019; LBP, Lebanese pounds; BRS, Brief resilience scale; CI, Confidence interval.

## Data Availability

The datasets used and/or analyzed during the current study are available from the corresponding author on reasonable request. The online questionnaire used for data collection is available in Supplementary Material File S1.

## Supplementary Materials

The following supporting information can be downloaded at: https://www.mdpi.com/article/10.3390/ejihpe12080077/s1.

## References

[1] Huang C., Wang Y., Li X., Ren L., Zhao J., Hu Y., Zhang L., Fan G., Xu J., Gu X. (2020). Clinical features of patients infected with 2019 novel coronavirus in Wuhan, China. Lancet.

[2] WHO Director-General’s Opening Remarks at the Media Briefing on COVID-19. 11 March 2020. https://www.who.int/dg/speeches/detail/who-director-general-s-opening-remarks-at-the-media-briefing-on-covid-19---11-march-2020.

[3] Barbuddhe S.B., Rawool D.B., Gaonkar P.P., Vergis J., Dhama K., Malik S.S. (2020). Global scenario, public health concerns and mitigation strategies to counter current ongoing SARS-CoV-2/COVID-19 pandemic. Hum. Vaccines Immunother..

[4] Benach J. (2021). We Must Take Advantage of This Pandemic to Make a Radical Social Change: The Coronavirus as a Global Health, Inequality, and Eco-Social Problem. Int. J. Health Serv..

[5] Mental Health and Psychosocial Considerations during the COVID-19 Outbreak. https://www.who.int/publications-detail-redirect/WHO-2019-nCoV-MentalHealth-2020.1.

[6] Shuja K.H., Aqeel M., Jaffar A., Ahmed A. (2020). COVID-19 Pandemic and Impending Global Mental Health Implications. Psychiatr. Danub..

[7] Torales J., O’Higgins M., Castaldelli-Maia J.M., Ventriglio A. (2020). The outbreak of COVID-19 coronavirus and its impact on global mental health. Int. J. Soc. Psychiatry.

[8] Brooks S.K., Webster R.K., Smith L.E., Woodland L., Wessely S., Greenberg N., Rubin G.J. (2020). The psychological impact of quarantine and how to reduce it: Rapid review of the evidence. Lancet.

[9] Fruehwirth J.C., Biswas S., Perreira K.M. (2021). The COVID-19 pandemic and mental health of first-year college students: Examining the effect of COVID-19 stressors using longitudinal data. PLoS ONE.

[10] Huckins J.F., da Silva A.W., Wang W., Hedlund E., Rogers C., Nepal S.K., Wu J., Obuchi M., Murphy E.I., Meyer M.L. (2020). Mental Health and Behavior of College Students During the Early Phases of the COVID-19 Pandemic: Longitudinal Smartphone and Ecological Momentary Assessment Study. J. Med. Internet Res..

[11] Mack D.L., DaSilva A.W., Rogers C., Hedlund E., Murphy E.I., Vojdanovski V., Plomp J., Wang W., Nepal S.K., Holtzheimer P.E. (2021). Mental Health and Behavior of College Students During the COVID-19 Pandemic: Longitudinal Mobile Smartphone and Ecological Momentary Assessment Study, Part II. J. Med. Internet Res..

[12] Sahu P. (2020). Closure of Universities Due to Coronavirus Disease 2019 (COVID-19): Impact on Education and Mental Health of Students and Academic Staff. Cureus.

[13] Ma Z., Zhao J., Li Y., Chen D., Wang T., Zhang Z., Chen Z., Yu Q., Jiang J., Fan F. (2020). Mental health problems and correlates among 746 217 college students during the coronavirus disease 2019 outbreak in China. Epidemiol. Psychiatr. Sci..

[14] Wang X., Hegde S., Son C., Keller B., Smith A., Sasangohar F. (2020). Investigating Mental Health of US College Students During the COVID-19 Pandemic: Cross-Sectional Survey Study. J. Med. Internet Res..

[15] Pramukti I., Strong C., Sitthimongkol Y., Setiawan A., Pandin M.G.R., Yen C.-F., Lin C.-Y., Griffiths M.D., Ko N.-Y. (2020). Anxiety and Suicidal Thoughts During the COVID-19 Pandemic: Cross-Country Comparative Study Among Indonesian, Taiwanese, and Thai University Students. J. Med. Internet Res..

[16] Kecojevic A., Basch C.H., Sullivan M., Davi N.K. (2020). The impact of the COVID-19 epidemic on mental health of undergraduate students in New Jersey, cross-sectional study. PLoS ONE.

[17] Wathelet M., Duhem S., Vaiva G., Baubet T., Habran E., Veerapa E., Debien C., Molenda S., Horn M., Grandgenèvre P. (2020). Factors Associated with Mental Health Disorders Among University Students in France Confined During the COVID-19 Pandemic. JAMA Netw. Open.

[18] (2019). Coronavirus Disease (COVID-2019) National Health Strategic Preparedness and Response Plan. http://www.moph.gov.lb/en/Media/view/27426/coronavirus-disease-health-strategic-preparedness-and-response-plan-.

[19] Halat D.H., Akel M., Hajj F., HajjHussein H., Kansoun R., Sharif-Askari E., Siblani L., Faraj A. (2020). Insights into the Positive Role of a Higher Education Institution in the Prevention of Misinformation During Pandemics: The Health Committee Model During COVID-19. Coronaviruses.

[20] Monitoring of COVID-19 Infection in Lebanon. 22 July 2022. http://www.moph.gov.lb/en/Media/view/43750/1/monitoring-of-covid-19-.

[21] Hallal K., HajjHussein H., Tlais S. (2020). A Quick Shift from Classroom to Google Classroom: SWOT Analysis. J. Chem. Educ..

[22] Halat D.H., Cherfan M., Mourad N., Rahal M. (2020). Highlights from a model for remote delivery of pharmacy laboratory courses: Design, implementation and student feedback: Innovation in learning assessment. Pharm. Educ..

[23] Halat D.H., Safwan J., Akel M., Rahal M. (2022). PROGRAMME DESCRIPTION: Pharmacy education shift during times of pandemic and collapse: A perspective from a school of pharmacy in Lebanon. Pharm. Educ..

[24] Sabourin A.A., Prater J.C., Mason N.A. (2019). Assessment of mental health in doctor of pharmacy students. Curr. Pharm. Teach. Learn..

[25] Votta R.J., Benau E.M. (2014). Sources of stress for pharmacy students in a nationwide sample. Curr. Pharm. Teach. Learn..

[26] Fischbein R., Bonfine N. (2019). Pharmacy and Medical Students’ Mental Health Symptoms, Experiences, Attitudes and Help-Seeking Behaviors. Am. J. Pharm. Educ..

[27] Yousif M.A., Arbab A.H., Yousef B.A. (2022). Perceived Academic Stress, Causes, and Coping Strategies Among Undergraduate Pharmacy Students During the COVID-19 Pandemic. Adv. Med. Educ. Pract..

[28] Alomar M., Palaian S., Shanableh S. (2021). Perceived Stress and Quality of Life Among Final-Year Pharmacy Students in the United Arab Emirates During COVID-19 Pandemic Lockdown. Adv. Med. Educ. Pract..

[29] Davis E.J., Amorim G., Dahn B., Moon T.D. (2021). Perceived ability to comply with national COVID-19 mitigation strategies and their impact on household finances, food security, and mental well-being of medical and pharmacy students in Liberia. PLoS ONE.

[30] Campos J.A.D.B., Campos L.A., Bueno J.L., Martins B.G. (2021). Emotions and mood swings of pharmacy students in the context of the coronavirus disease of 2019 pandemic. Curr. Pharm. Teach. Learn..

[31] El Othman R., Touma E., El Othman R., Haddad C., Hallit R., Obeid S., Salameh P., Hallit S. (2021). COVID-19 pandemic and mental health in Lebanon: A cross-sectional study. Int. J. Psychiatry Clin. Pract..

[32] Msheik El Khoury F., Talih F., Khatib M.F.E., Abi Younes N., Siddik M., Siddik-Sayyid S. (2021). Factors Associated with Mental Health Outcomes: Results from a Tertiary Referral Hospital in Lebanon during the COVID-19 Pandemic. Libyan J. Med..

[33] Fawaz M., Samaha A. (2021). E-learning: Depression, anxiety, and stress symptomatology among Lebanese university students during COVID-19 quarantine. Nurs. Forum.

[34] Salameh P., Hajj A., Badro D.A., Abou Selwan C., Aoun R., Sacre H. (2020). Mental Health Outcomes of the COVID-19 Pandemic and a Collapsing Economy: Perspectives from a Developing Country. Psychiatry Res..

[35] Tran T.D., Tran T., Fisher J. (2013). Validation of the depression anxiety stress scales (DASS) 21 as a screening instrument for depression and anxiety in a rural community-based cohort of northern Vietnamese women. BMC Psychiatry.

[36] Le M.T.H., Tran T.D., Holton S., Nguyen H.T., Wolfe R., Fisher J. (2017). Reliability, convergent validity and factor structure of the DASS-21 in a sample of Vietnamese adolescents. PLoS ONE.

[37] Rutter M. (2006). Implications of resilience concepts for scientific understanding. Ann. N. Y. Acad. Sci..

[38] Smith B.W., Dalen J., Wiggins K., Tooley E., Christopher P., Bernard J. (2008). The brief resilience scale: Assessing the ability to bounce back. Int. J. Behav. Med..

[39] Fouad F.M., Barkil-Oteo A., Diab J.L. (2020). Mental Health in Lebanon’s Triple-Fold Crisis: The Case of Refugees and Vulnerable Groups in Times of COVID-19. Front. Public Health.

[40] Tan B.Y.Q., Chew N.W.S., Lee G.K.H., Jing M., Goh Y., Yeo L.L.L., Zhang K., Chin H.-K., Ahmad A., Khan F.A. (2020). Psychological Impact of the COVID-19 Pandemic on Health Care Workers in Singapore. Ann. Intern. Med..

[41] Yıldırım M., Arslan G. (2020). Exploring the associations between resilience, dispositional hope, preventive behaviours, subjective well-being, and psychological health among adults during early stage of COVID-19. Curr. Psychol..

[42] Lindinger-Sternart S., Kaur V., Widyaningsih Y., Patel A.K. (2021). COVID-19 phobia across the world: Impact of resilience on COVID-19 phobia in different nations. Couns Psychother. Res..

[43] Abed A.E., Razzak R.A., Hashim H.T. (2021). Mental Health Effects of COVID-19 within the Socioeconomic Crisis and After the Beirut Blast Among Health Care Workers and Medical Students in Lebanon. Prim. Care Companion CNS Disord..

[44] Basheti I.A., Mhaidat Q.N., Mhaidat H.N. (2021). Prevalence of anxiety and depression during COVID-19 pandemic among healthcare students in Jordan and its effect on their learning process: A national survey. PLoS ONE.

[45] Hossain M.M., Tasnim S., Sultana A., Faizah F., Mazumder H., Zou L., McKyer E.L.J., Ahmed H.U., Ma P. (2020). Epidemiology of mental health problems in COVID-19: A review. F1000Research.

[46] Holmes E.A., O’Connor R.C., Perry V.H., Tracey I., Wessely S., Arseneault L., Ballard C., Christensen H., Silver R.C., Everall I. (2020). Multidisciplinary research priorities for the COVID-19 pandemic: A call for action for mental health science. Lancet Psychiatry.

[47] Rajab M.H., Gazal A.M., Alkattan K. (2020). Challenges to Online Medical Education During the COVID-19 Pandemic. Cureus.

[48] Baloch G.M., Sundarasen S., Chinna K., Nurunnabi M., Kamaludin K., Khoshaim H.B., Hossain S.F.A., AlSukayt A. (2021). COVID-19: Exploring impacts of the pandemic and lockdown on mental health of Pakistani students. PeerJ.

[49] Silva R.G., Figueiredo-Braga M. (2018). Evaluation of the relationships among happiness, stress, anxiety, and depression in pharmacy students. Curr. Pharm. Teach. Learn..

[50] Cho E., Jeon S. (2019). The role of empathy and psychological need satisfaction in pharmacy students’ burnout and well-being. BMC Med. Educ..

[51] Hallit S., Sacre H., Hajj A., Sili G., Zeenny R.M., Salameh P. (2019). Projecting the future size of the Lebanese pharmacy workforce: Forecasts until the year 2050. Int. J. Pharm. Pract..

[52] Younes S., Safwan J., Rahal M., Hammoudi D., Akiki Z., Akel M. (2021). Effect of COVID-19 on mental health among the young population in Lebanon. Encephale.

[53] Elsalem L., Al-Azzam N., Jum’ah A.A., Obeidat N., Sindiani A.M., Kheirallah K.A. (2020). Stress and behavioral changes with remote E-exams during the COVID-19 pandemic: A cross-sectional study among undergraduates of medical sciences. Ann. Med. Surg..

[54] Nouira H., Ben Abdelaziz A., Rouis S., Mili M., Safer M., Ben Saad H., Ben Abdelaziz A. (2018). Smoking behavior among students of health sciences at the university of Monastir (Tunisia). Tunis Med..

[55] Ilic I., Grujicic Sipetic S., Radovanovic D., Ilic M. (2020). Cigarette Smoking and E-Cigarette Use by Pharmacy Students in Serbia. Behav. Med..

[56] Jradi H., Wewers M.E., Pirie P.R., Binkley P.F., Ferketich K. (2013). Cigarette and waterpipe smoking associated knowledge and behaviour among medical students in Lebanon. East Mediterr. Health J..

[57] Alqahtani J.S., Oyelade T., Aldhahir A.M., Alghamdi S.M., Almehmadi M., Alqahtani A.S., Quaderi S., Mandal S., Hurst J.R. (2020). Prevalence, Severity and Mortality associated with COPD and Smoking in patients with COVID-19: A Rapid Systematic Review and Meta-Analysis. PLoS ONE.

[58] Odriozola-González P., Planchuelo-Gómez Á., Irurtia M.J., de Luis-García R. (2020). Psychological effects of the COVID-19 outbreak and lockdown among students and workers of a Spanish university. Psychiatry Res..

[59] Sepúlveda-Loyola W., Rodríguez-Sánchez I., Pérez-Rodríguez P., Ganz F., Torralba R., Oliveira D.V., Rodríguez-Mañas L. (2020). Impact of Social Isolation Due to COVID-19 on Health in Older People: Mental and Physical Effects and Recommendations. J. Nutr. Health Aging.

[60] Tak C., Henchey C., Feehan M., Munger M.A. (2019). Modeling Doctor of Pharmacy Students’ Stress, Satisfaction, and Professionalism Over Time. Am. J. Pharm. Educ..

[61] Capurro G., Jardine C.G., Tustin J., Driedger M. (2021). Communicating scientific uncertainty in a rapidly evolving situation: A framing analysis of Canadian coverage in early days of COVID-19. BMC Public Health.

[62] Singh R., Mahato S., Singh B., Thapa J., Gartland D. (2019). Resilience In Nepalese Adolescents: Socio-Demographic Factors Associated with Low Resilience. J. Multidiscip. Healthc..

[63] Robertson H.D., Elliott A.M., Burton C., Iversen L., Murchie P., Porteous T., Matheson C. (2016). Resilience of primary healthcare professionals: A systematic review. Br. J. Gen. Pract..

[64] Raghunathan S., Darshan Singh A., Sharma B. (2022). Study of Resilience in Learning Environments During the Covid-19 Pandemic. Front. Educ..

[65] Cosco T.D., Cooper R., Kuh D., Stafford M. (2018). Socioeconomic inequalities in resilience and vulnerability among older adults: A population-based birth cohort analysis. Int. Psychogeriatr..

[66] Calo M., Peiris C., Chipchase L., Blackstock F., Judd B. (2019). Grit, resilience and mindset in health students. Clin. Teach..

[67] Skalski S.B., Konaszewski K., Büssing A., Surzykiewicz J. (2022). Resilience and Mental Well-Being During the COVID-19 Pandemic: Serial Mediation by Persistent Thinking and Anxiety About Coronavirus. Front. Psychiatry.

[68] Appolloni A., Colasanti N., Fantauzzi C., Fiorani G., Frondizi R. (2021). Distance Learning as a Resilience Strategy during COVID-19: An Analysis of the Italian Context. Sustainability.

[69] Durbas A., Karaman H., Solman C.H., Kaygisiz N., Ersoy Ö. (2021). Anxiety and Stress Levels Associated with COVID-19 Pandemic of University Students in Turkey: A Year After the Pandemic. Front. Psychiatry.

[70] Smith K., Lambe S., Freeman D., Cipriani A. (2021). COVID-19 vaccines, hesitancy and mental health. Evid. Based Ment. Health.

