# Supplementary material for: Pharmacy Students’ Mental Health and Resilience in COVID-19: An Assessment after One Year of Online Education
Source: Eur J Investig Health Psychol Educ. 2022 Aug 15;12(8):1082–107. doi: 10.3390/ejihpe12080077 (PMC9407568; doi:10.3390/ejihpe12080077)
Supplement: Supplementary file 1 [file ejihpe-12-00077-s001.zip › ejihpe-1802606-supplementary.pdf]

Supplementary Materials

This Supplementary Materials shows the online questionnaire used for data collection in this study. The questionnaire was converted to an online Google Form and sent to participants using their university emails and the Google Classroom platform.

COVID-19 Impact on Pharmacy Students' Mental Health After One Year

Dear Pharmacy Students,

Coronavirus disease 2019 (COVID-19) is a pandemic with devastating global effects. A little over one year after declaration of the pandemic by the World Health Organization, a state of physical distancing, lock-down and social/travel restrictions is still affecting most countries. The psychological impact of the pandemic on mental health of different populations, including university students, warrants study and assessment.

This survey was developed by a group of researchers at the Lebanese International University to assess the impact of COVID-19 on mental health of our pharmacy students one year following declaration of the pandemic and after almost three semesters of pharmacy education delivered remotely.

We are grateful for your cooperation in answering this survey. Participation is absolutely voluntary, and your responses shall remain anonymous and con1dential. Completion of the survey till the end is considered as informed consent to participate.

Thank you,
The Research Team

**A. Demographic Data**		
A1. Gender<br>• Male<br>• Female<br>A2. Age:<br>• ------ years<br>A3. Nationality<br>• Lebanese<br>• Non-Lebanese<br>A4. Area of Residence<br>• Bekaa<br>• Baalbeck/Hermel<br>• Mount Lebanon<br>• Beirut<br>• North<br>• South<br>• Akkar<br>• Nabatieh<br>• I currently reside outside Lebanon<br>A5. Marital Status<br>• Single<br>• Married<br>• Divorced/Widowed<br>A6. Do you have any children?<br>• Yes<br>• No<br>A7. Household Size<br>• One person<br>• 2 people<br>• 3-5 people<br>• 6 people or more	A8. Year in pharmacy school<br>• First<br>• Second<br>• Third<br>• Fourth<br>• Fifth<br>• PharmD<br>A9. Family income per month<br>• 2,000,000 LBP or less<br>• 2,000,001-4,000,000 LBP<br>• 4,000,001-6,000,000 LBP<br>• More than 6,000,000 LBP<br>A10. Has your family income been affected by lockdown due to COVID-19?<br>• Yes<br>• No<br>A11. Are you consuming alcohol during COVID-19?<br>• No<br>• Yes, once every few weeks<br>• Yes, once to twice per week<br>• Yes, more than twice per week<br>A12. Are you consuming caffeine during COVID-19?<br>• No<br>• Yes, less than 2 cups per day<br>• Yes, 2-4 cups per day<br>• Yes, more than 4 cups per day	A13. Are you smoking during COVID-19?<br>• No<br>• Yes, less than 10 cigarettes per day and/or less than 3 shisha per week<br>• Yes, 10 or more cigarettes per day and/or 3 or more shisha per week<br>• Other:<br>A14. Are you consuming energy drinks during COVID-19?<br>• No<br>• Yes, once per week<br>• Yes, more than once per week<br>A15. Are you taking any medications during COVID-19? *Check all that apply.*<br>• No<br>• Yes, acetaminophen<br>• Yes, NSAIDS<br>• Yes, antihistamines<br>• Yes, multivitamins and/or minerals<br>• Yes, antidepressants and/or anxiolytics<br>• Yes, others<br>A16. Has your body weight changed during COVID-19?<br>• No<br>• Yes, I gained weight<br>• Yes, I lost weight<br>A17. Are you or your close family receiving financial support in foreign currency * from family members or relatives who live outside Lebanon ?<br>• Yes<br>• No

Part B: DASS21 (Depression, Anxiety, Stress Scale - 21 Items) Assessment

Please read each statement and select a number 0, 1, 2 or 3 which indicates how much the statement applied to you over the past week. There are no right or wrong answers. Do not spend too much time on any statement.

PLEASE RATE AS FOLLOWS:

- 0 Did not apply to me at all – NEVER
- 1 Applied to me to some degree, or some of the time – SOMETIMES
- 2 Applied to me to a considerable degree, or a good part of time – OFTEN
- 3 Applied to me very much, or most of the time – ALMOST ALWAYS

	0 (NEVER)	1 (SOMETIMES)	2 (OFTEN)	3 (ALMOST ALWAYS)
1. I found it hard to wind down				
2. I was aware of dryness of my mouth				
3. I couldn't seem to experience any positive feeling at all				
4. I experienced breathing difficulty (eg, excessively rapid breathing, breathlessness in the absence of physical exertion)				
5. I found it difficult to work up the initiative to do things				
6. I tended to over-react to situations				
7. I experienced trembling (eg, in the hands)				
8. I felt that I was using a lot of nervous energy				
9. I was worried about situations in which I might panic and make a fool of myself				
10. I felt that I had nothing to look forward to				
11. I found myself getting agitated				
12. I found it difficult to relax				
13. I felt down-hearted and blue				
14. I was intolerant of anything that kept me from getting on with what I was doing				
15. I felt I was close to panic				
16. I was unable to become enthusiastic about anything				
17. I felt I wasn't worth much as a person				
18. I felt that I was rather touchy				
19. I was aware of the action of my heart in the absence of physical exertion (eg. sense of heart rate increase, heart missing a beat)				
20. I felt scared without any good reason				
21. I felt that life was meaningless				

Part C: Brief Resilience Scale

Please indicate the extent to which you agree with each of the following statements by using the following scale: 1 = strongly disagree, 2 = disagree, 3 = neutral, 4 = agree, 5 = strongly agree.

	1 (strongly disagree)	2 (disagree)	3 (neutral)	4 (agree)	5 (strongly agree)
1. I tend to bounce back quickly after hard times					
2. I have a hard time making it through stressful events					
3. It does not take me long to recover from a stressful event					
4. It is hard for me to snap back when something bad happens					
5. I usually come through di2cult times with little trouble					
6. I tend to take a long time to get over set-backs in my life					

Part D: COVID- 19 Stressors

This part of the survey assesses economic, educational, and health stressors due to COVID-19.

D1. Are you employed?
- Yes
- No

D2. Have you lost your employment during COVID-19?
- Yes
- No
- Does not apply

D3. Have you experienced employment reduction during COVID-19?
- Yes
- No
- Does not apply

D4. Have any of your parents lost employment during COVID-19?
- Yes
- No

D5. Have any of your parents experienced a reduction in salary or income during COVID-19?
- Yes
- No

D7. Have you been diagnosed with COVID-19?
- No
- Yes, I had mild to moderate symptoms
- Yes, I had moderate to severe symptoms
- Yes, I was hospitalized

D8. Has any of your close family members (in the same household) been diagnosed with COVID-19?
- No
- Yes, with mild to moderate symptoms
- Yes, with moderate to severe symptoms
- Yes, and was hospitalized

D9. Do you feel isolated from others due to COVID-19?
- Always
- Usually
- Rarely
- Never

D6. Please rate the following regarding distance learning at the school of pharmacy during COVID-19 on a scale from 1 to 5: 1 = strongly disagree, 2 = disagree, 3 = neutral, 4 = agree, 5 = strongly agree.

	1 (strongly disagree)	2 (disagree)	3 (neutral)	4 (agree)	5 (strongly agree)
1. I am satisfied with distance learning of theoretical pharmacy courses.					
2. I am satisfied with delivery of laboratory courses, simulation, and practice experience remotely.					
3. I am satisfied with the use of technology for distance learning (Google classroom, Zoom, Google forms, Moodle, etc..).					
4. I am overall satisfied with my exam performance and grades during distance learning.					
5. I am satisfied with communication with my instructors and colleagues during distance learning.					

Part E: Vaccine Attitudes

E1. Have you received any COVID-19 vaccine?
- Yes *Skip to question E1a*
- No *Skip to question E1b*

E1a - Vaccinated

E1. Which vaccine did you receive?
- Pfizer -BioNTech vaccine
- Oxford/AstraZeneca vaccine
- Sinopharm vaccine
- Sinovac vaccine
- Sputnik V vaccine

E2. Do you trust COVID-19 vaccine benefits and the role of vaccines in controlling the pandemic?
- Yes
- No
- Somehow

E3. Are you worried about the side effects of COVID-19 vaccines?
- Yes
- No
- Somehow

E4. Do you trust the governmental plan in distribution and administration of COVID-19 vaccines?
- Yes
- No
- Somehow

E1b - Not Vaccinated

E1. Do you plan to get vaccinated when you become eligible for COVID-19 vaccine?
- Yes
- No
- Maybe

E2. Do you trust COVID-19 vaccine benefits and the role of vaccines in controlling the pandemic?
- Yes
- No
- Somehow

E3. Are you worried about the side effects of COVID-19 vaccines?
- Yes
- No
- Somehow

E4. Do you trust the governmental plan in distribution and administration of COVID-19 vaccines?
- Yes
- No
- Somehow

Thank you for Completing this Questionnaire.
